# Daily Affect Dynamics Mediate the Longitudinal and Reciprocal Link Between Depressive Symptoms and Inflammation

**DOI:** 10.1093/geroni/igaf122.823

**Published:** 2025-12-31

**Authors:** Sun Ah Lee, David Almeida

**Affiliations:** The Pennsylvania State University, University Park, Pennsylvania, United States; The Pennsylvania State University, University Park, Pennsylvania, United States

## Abstract

Depression is a leading mental health concern among middle-aged and older adults. Extensive research has established an intricate relationship between depression and inflammation, yet greater attention is needed to elucidate the mechanisms through which these processes unfold across multiple time scales and levels of analysis. This study evaluated the mediating role of daily affect dynamics in the bidirectional and longitudinal association between depressive symptoms and inflammation. Using data from the second and third waves of the Midlife in the United States (MIDUS) study, the sample included 563 adults who participated in both the Daily Diary and Biomarker Projects (Mage = 52.4; 57% female; 84% White). Daily affect and stress were measured over eight consecutive days. Depressive symptoms were assessed using the CESD-20. Inflammatory markers were used to create two separate indicators: composite cytokine scores (IL-6, IL-8, TNFα, IL-10) and CRP, derived from fasting blood samples. Multilevel structural equation models simultaneously tested between-person autoregressive and cross-lagged paths of the depressive symptoms-inflammation link, as well as within-person mediated paths involving daily affect dynamic indicators. Results revealed that positive affective variability and negative affective reactivity to daily stressors significantly mediated the associations between depressive symptoms and CRP. These patterns were more evident among middle-aged than older adults. Between-person autoregressive paths for depressive symptoms and inflammation were significant, whereas cross-lagged paths showed no significant associations. Findings suggest daily affective processes as potential mechanisms linking mental and physical health, underscoring the need for interventions that promote affective stability and adaptive stress responses in everyday life.

